# The relationship between teachers' information technology integration self-efficacy and TPACK: A meta-analysis

**DOI:** 10.3389/fpsyg.2022.1091017

**Published:** 2022-12-02

**Authors:** Youlai Zeng, Yue Wang, Shunyu Li

**Affiliations:** ^1^Department of Education, School of Education, Liaoning Normal University, Dalian, China; ^2^Center for Teacher Education Research in Xinjiang, School of Educational Sciences, Xinjiang Normal University, Wulumuqi, China

**Keywords:** TPACK, information technology integration self-efficacy, meta-analysis, teacher, moderating variables

## Abstract

Using the method of meta-analysis, this study explored the relationship between teachers' self-efficacy and TPACK in the context of educational information technology integration and focused on the moderating variables that affect the relationship. Through literature search, 28 independent effect sizes with 7,777 subjects were obtained. Heterogeneity test illustrated that random effects model is appropriate. Funnel plot and Begg and Mazumdar's rank correlation test found there was no publication bias in this meta-analysis. After effect size test, it followed that teachers' information technology integration self-efficacy was significantly positively correlated with TPCK (r = 0.607, *P* < 0.001). The moderating effect test indicated that the relationship was moderated by the subjects' career stages, but not by gender, teaching stages, disciplines, and measurement tools.

## Introduction

Teachers need to have a rich conceptual understanding of the teaching contents, and combine them with teaching professional knowledge such as teaching procedures, teaching strategies, and teaching methods. After continuous development, application, and adjustment, it can be formed Pedagogical Content Knowledge defined by Shulman (1987). The theoretical creation and development of Pedagogical Content Knowledge laid the foundation for the theoretical construction of technology pedagogical and content knowledge (TPACK) by Koehler & Mishra and others. Subsequently, the TPACK questionnaire developed by Schmidt provided measurement tools for relevant empirical research, marking the development and maturity of TPACK theory, and it also attracted more attention to the research on integrating information technology into education and teaching. Although information technology provides good conditions for teaching to create interactive activities, the use of information technology has not had a positive impact on teaching quality. Teachers' knowledge system has undergone a structural change is of positive significance under the background of information technology integration. That is, from content knowledge, pedagogical knowledge extending to technological pedagogical and content knowledge.

Through the analysis of TPACK research literature in the past 16 years, it assumed that information technology integration self-efficacy is one of the important influencing factors of teachers' TPACK. Holland constructed the technology integration education Model (TIE Model) and investigated the influencing factors of teachers' TPACK (Holland and Piper, 2014). The impact of values, attitudes, self-confidence and self-efficacy of information technology integration has been confirmed (Koçak-Usluel et al., 2015). As Ertmer pointed out, if teachers do not have a strong sense of information technology integration self-efficacy, they can not make full use of information technology to integrate knowledge and teaching skills (Ertmer and Ottenbreit-Leftwich, 2010). At present, few scholars systematically analyze the quantitative relationship between teachers' information technology integration self-efficacy and teachers' knowledge. Accordingly, this study adopted the method of meta-analysis to examine the relationship between them and explore the moderating variables that may affect the relationship, in order to draw a more general and scientific conclusion.

## Concept and measurement of information technology integration self-efficacy

Bandura considered when individuals make judgments that they are capable of completing a certain task, their motivation will be enhanced (Bandura, 1982). Self-efficacy referred to beliefs in the abilities that individuals need to organize and implement plans to achieve specific goals (Bandura, 1977). In the field of education research, Bandura's operational definition of teachers' self-efficacy includes seven dimensions: decision-making efficiency; school resource efficiency; teaching efficiency; discipline maintenance efficiency; parental participation efficiency; community participation efficiency; campus atmosphere creation efficiency. Later, Tschannen-Moran and other scholars have revised the definition, which defined as teachers' belief in the organizational and executive ability required for them to successfully complete specific teaching tasks and action processes under a certain background (Tschannen-Moran et al., 1998). In the field of information technology research, based on Bandura's self-efficacy theory, computer self-efficacy (Murphy et al., 1989; Compeau and Higgins, 1995), Internet self-efficacy (Torkzadeh and Van Dyke, 2001; Hsu and Chiu, 2004), and other concepts were put forward one after another, and then relevant measurement tools and scales were also examined and applied.

Wang contended teachers' information technology integration self-efficacy is a perception of the ability to effectively master and use technologies (such as computer, Internet, and multimedia, etc.) to achieve specific teaching objectives (Wang and Zhao, 2021). As information technology is embedded in education and teaching, more and more researches have been conducted on teachers' information technology integration self-efficacy, and the measurement tools aimed at evaluating the ability of teachers to complete expected information technology-related actions in education and teaching are also diversified. The first type is a specially developed measurement tools of information technology integration self-efficacy. Scale compiled by Wang included 16 items, and the pre-test and post-test reliability coefficients are 0.94 and 0.96, respectively (Wang et al., 2004). Since then, the scale has been widely used in the empirical study of teachers' information technology integration self-efficacy (Niederhauser and Perkmen, 2008; Abbitt, 2011; Zahwa et al., 2021), and got further revised and simplified (Perkmen, 2008; Yeh et al., 2021). The second type is measurement tools adapted from the computer self-efficacy and Internet self-efficacy scale, with only partial adjustments in statements (Sahin et al., 2013; López-Vargas et al., 2017; Cai et al., 2019). Blonder developed a questionnaire of high school chemistry teachers self-efficacy in the context of Facebook use, which was adapted from the teachers self-efficacy questionnaire (Tschannen-Moran and Woolfolk Hoy, 2001) and the computer self-efficacy questionnaire (Murphy et al., 1989), consisting of Facebook use self-efficacy and Facebook uses self-efficacy in teaching (Blonder and Rap, 2017). The last type is to directly use the teacher's self-efficacy scale for measurement, but emphasize the background of the use of information technology (Mishne, 2012).

## The relationship between information technology integration self-efficacy and TPACK

TPACK is a developmental teacher knowledge structure, which also represents the ability and level of the teacher's information technology integration behavior in class. In 2005, Mishra and Koehler integrated technology into pedagogical content knowledge (PCK), first proposed the concept of technological pedagogical content knowledge (TPCK) (Koehler and Mishra, 2005), and elaborated the constituent elements of TPCK in 2006 (Mishra and Koehler, 2006). In 2007, Thompson and Mishra renamed it TPACK (Technological Pedagogical and Content Knowledge, pronounced “tee-pack”), meaning Total PACKage. It further emphasizes the importance of integrating content knowledge, pedagogical knowledge and technology knowledge (Thompson and Mishra, 2007; Tseng et al., 2022). TPACK includes 7 elements in total: Content Knowledge (CK), Pedagogical Knowledge (PK), Technology knowledge (TK), and other elements are formed by the interaction between the former, namely, Pedagogical Content Knowledge (PCK), Technological Content Knowledge (TCK), Technological Pedagogical Knowledge (TPK), Technological Pedagogical Content Knowledge (TPCK). Therefore, the effective generation of teaching behavior integrated information technology requires teachers to continuously create, maintain and rebuild the dynamic balance between 7 elements, which needs teachers to clearly select what technology to choose and how to integrate it in teaching (Koehler and Mishra, 2009; Almaiah et al., 2022). Information technology integration behavior is a key representation of TPACK ability. More specifically, teachers' belief about their ability to successfully implement information technology integration behavior is closely related to TPACK, and it is one of the vital factors affecting decision-making and the use of technology in class (Abbitt, 2011). The research has confirmed that when teachers have a strong belief in information technology integration and believe that learning and using technology is interesting, important and useful, they will have a stronger motivation to improve the ability of information technology integration, and then obtain a higher level of TPACK (Anderson and Maninger, 2007; Hsu et al., 2017; König et al., 2022). Conversely, teachers with high TPACK levels but low information technology integration self-efficacy, perhaps, do not use these skills effectively (Kaşci and Selçuk, 2021).

TPACK and information technology integration self-efficacy interact with each other, and this interaction also change with research background. In other words, the interaction between them is dynamic, changing and developing. Different research objectives, tools, statistical methods and other factors may lead to different relationships. The review of empirical research literature shows that, on the one hand, TPACK has a positive impact on teachers' information technology integration self-efficacy (Joo et al., 2018; Yang, 2018; Wang, 2020, 2022; Durak, 2021), however, in terms of various dimensions of TPACK, there are certain differences in this relationship. Abbitt found that TPK, PCK, and TK had a significant positive effect on teachers' information technology integration self-efficacy, but PK had a negative effect on it, and TCK and TPCK had no significant effect (Abbitt, 2011). On the other hand, the positive effect of teachers' information technology integration self-efficacy on TPACK has also been proved, namely, the stronger self-efficacy teachers are, the higher TPACK level they get (Semiz and Ince, 2012; He, 2013; Cao, 2016; Chen, 2018; Xu, 2020; Ladendorf et al., 2021). Cai's research on flipped classroom teaching in universities demonstrated that teachers' computer self-efficacy has a positive effect on their TPCK, and this conclusion was also verified in the research on primary and secondary school teachers (Dong et al., 2020). However, the impact of information technology integration self-efficacy on the elements of TPACK is inconsistent. Song found that teachers' information technology self-efficacy has a direct impact on TPK, but has no direct impact on TPCK in the use of digital textbooks (Song and Sun, 2014). In summary, most scholars have confirmed that there is a significant relationship between teachers' information technology integration self-efficacy and TPACK (Oskay, 2017; Tondeur et al., 2017b; Wang and Zhao, 2021), but in all dimensions of TPACK, this relationship is not completely significant (He, 2013; Lachner et al., 2021). Consequently, this study speculated that there are moderating variables in the relationship between teachers' information technology integration self-efficacy and TPACK.

## Moderating variables of the relationship between information technology integration self-efficacy and TPACK

### Gender

Current research on the gender effect of information technology self-efficacy has not drawn consistent conclusions. Studies have manifested that there is no differences in teachers' information technology integration self-efficacy between different genders (Bursal and Yigit, 2012; Chen, 2021), instead, some studies have shown that men's information technology integration self-efficacy is significantly higher than that of women (Vekiri and Chronaki, 2008; Ifinedo et al., 2020). Similarly, the research on the gender effect of TPACK sub-dimension has not reached a consistent conclusion. Some studies have shown that there is no significant difference in TPACK sub-dimension between teachers of different genders (Karalar and Altan, 2016). However, other studies have shown that there are gender differences in TPACK and its dimensions, that is, female teachers' CK and PCK are significantly higher than that of male teachers (Farrell and Hamed, 2017), while TK is significantly lower than male teachers (Lin et al., 2013; Ekrem and Recep, 2014). Chen found that there are significant gender differences in the overall level of teachers' TPACK, but there is no gender difference in the dimensions of TPK, TCK, and TPCK. In this case, is the relationship between teachers' information technology integration self-efficacy and TPACK also affected by gender? In the past, studies have rarely discussed it. Thus, this study used meta-analysis to explore the moderating effect of gender on the relationship between teachers' information technology integration self-efficacy and TPACK. This study proposed the first hypothesis that gender moderates the relationship between teachers' information technology integration self-efficacy and TPACK.

### Measurement tools

In the literature included in meta-analysis, the measurement tools of teachers' information technology integration self-efficacy fall into the following four categories: (1) Measurement tools were specially developed for information technology integration self-efficacy. The TISE compiled by Wang not only measured teachers' beliefs in technology ability, but also teachers' technology use strategies. Perkmen revised it and finally formed a simplified scale composed of 6 items, which is widely cited in academia. The Information Communications Technology Self-Efficacy (ICTSE) scale designed by Tondeur includes two dimensions: the ability to guide students to use information technology in class and the ability to use information technology in instructional design, consisting of 19 items. This scale focuses on the specific context of information technology use and its related strategies (Tondeur et al., 2017a). (2) Measurement tools were adapted from computer self-efficacy. The original computer self-efficacy scale (CSE) was compiled by Murphy and others, including three dimensions: primary skills, advanced skills and host skills, with 32 items in total (Murphy et al., 1989). Compeau held computer self-efficacy that refers to a judgment of one's capability to use a computer, and underlines the computer ability in the context of completing tasks. For example, “if someone guides me step by step” “I could use software to complete this work,” the participants can make a degree judgment from 10 items. Cai replaced “I could use software to complete this work” with “I could teach using flipped classroom Instruction” to conduct empirical research. (3) Teachers' self-efficacy scale was used to measure information technology integration self-efficacy. Some scholars directly use teachers' self-efficacy scale (TSE) (Tschannen-Moran and Woolfolk Hoy, 2001), but they emphasize the practical background of information technology integrated into teaching (Mishne, 2012; Song and Sun, 2014; Joo et al., 2018; Kaşci and Selçuk, 2021). This type of measurement method aims to evaluate teachers' beliefs in their own abilities and accomplishments, which is broader than directly focusing on information technology integration self-efficacy. In short, the focus of teachers' information technology self-efficacy measurement tools is various. This meta-analysis put forward the second research hypothesis that measurement tools have a moderating effect on the relationship between teachers' information technology integration self-efficacy and TPACK.

### Career stages, disciplines and teaching stages

In accordance with current literature, the subjects of the research mainly include pre-service teachers and in-service teachers. Most of the pre-service teachers are college students, who are receiving or have received systematic training in Technological Pedagogical and Content Knowledge, while in-service teachers often have rich teaching experience. So, we believe that these subjects probably have some differences in information technology integration self-efficacy and TPACK, and it is necessary to investigate the moderating role of career stages. Moreover, there are few studies on the effect of their teaching disciplines and teaching stages. Research revealed that each level of mathematics teachers' TPACK is significantly lower than that of English teachers and reading teachers, with the gap of 15% in TPCK (Farrell and Hamed, 2017); other studies concluded that science teachers' TK and TPCK are significantly higher than mathematics teachers (Jang and Tsai, 2012). Some scholars found there is significant difference in every dimensions of TPACK in different teaching stages (Li et al., 2022), but some other scholars believed that although primary school teachers do not have the same level of knowledge and skills compared with high school teachers, while their ability to integrate information technology into teaching practice may be similar (Farrell and Hamed, 2017). In the light of the previous research conclusions, there is no consistent conclusion about the differences between the effect of teaching subject and teaching stages. Accordingly, it is necessary to explore the moderating effect of the two on the relationship between teachers' information technology integration self-efficacy and TPACK, so as to draw a more general research conclusion. In conclusion, the current research mainly investigates the following two questions:

*First, what is the relationship between teachers' information technology integration self-efficacy and TPACK?*

*Second, is there a moderating effect on the relationship between information technology integration self-efficacy and TPACK from gender, measurement tools, career stages, disciplines and teaching stages on ?*

## Methods and materials

### Literature search and screening

#### Literature search

TPACK concept was formally proposed in 2007 (Thompson and Mishra, 2007), therefore, the year 2007 served as the starting point of our search until 2022. In order to retrieve the suitable and substantial quantitative empirical research studies, a systematic and comprehensive search was conducted, based on CNKI, Web of Science, SpringerLink, Google Scholar, and ProQuest. The main search terms are “self-efficacy” and “TPACK.”

#### Literature selection criteria

The inclusion criteria for the literature are as follows:

(1) Research topic: The topic is empirical research on the relationship between teachers' information technology integration self-efficacy and TPACK;
(2) Research method: The research method is quantitative empirical, excluding theoretical research or review articles;
(3) Research results: The literature clearly reports the measurement tools of information technology integration self-efficacy, including TISE, ICTSE, CSE, and TSE; The data are complete and clear, the correlation coefficient r between teachers' information technology integration self-efficacy and TPCK, or *F*-value, *t*-value, *X*^2^-value and other statistics which can be converted into r are clearly reported;
(4) Others: The repeated published articles are excluded, and the same data is used only once.

#### Literature screening process

**Figure 1:**
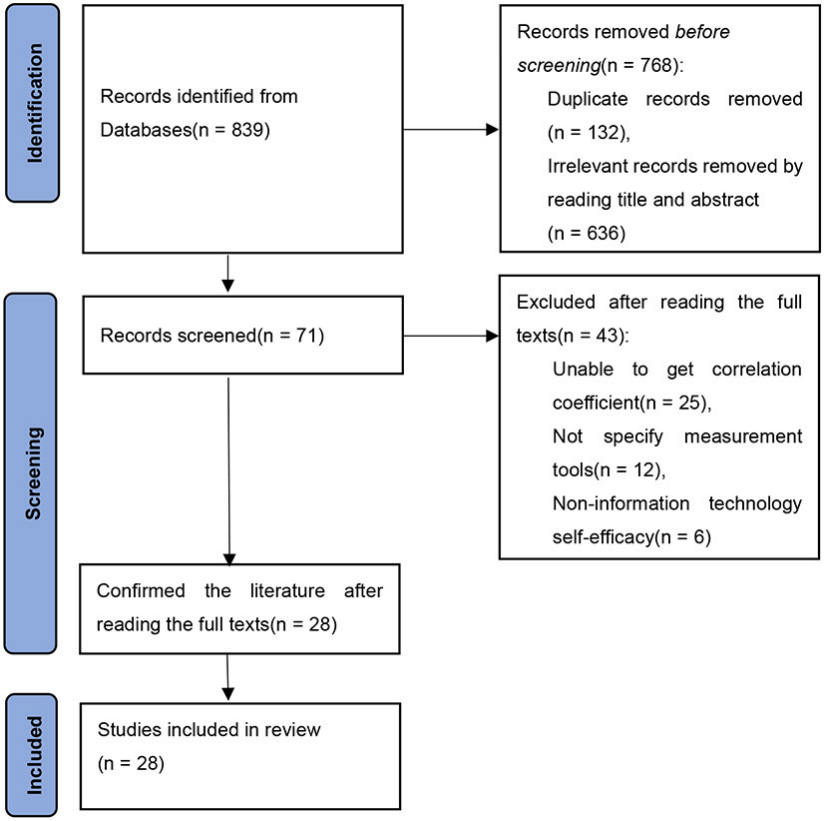
The PRISMA flow chart used to identify studies for detailed analysis of TPACK and information technology integration self-efficacy.

Literature screening was divided into three steps. The first step was identification, and a total of 839 papers were collected. After primary screening, 636 articles were eliminated by reading the titles and abstracts, and 132 duplicates were removed. The second step was screening. Forty-three articles were deleted based on the screening criteria. The third step was confirmation, through reading the full text again, 28 papers met the selection criteria, including 28 effect values. There are 8 Chinese articles in total, including 5 master's dissertations; and there are 20 English articles, including 1 doctoral dissertations and 1 special article in Indonesian with English abstracts. The flow chart of the article selection process is depicted in Figure 1.

### Document coding

The literature included in meta-analysis were coded as follows:

1. Literature information, including independent or first author, publication year;
2. Sample size;
3. Career stages, including pre-service and in-service;
4. Gender is expressed in male proportion;
5. Teaching periods including preschool, primary school, middle school, middle and primary school and university;
6. Teaching subjects, including general subject, English, mathematics, International Chinese, physical education, science, and chemistry;
7. Measurement tools, including TISE, ICTSE, CSE, and TSE;
8. Correlation coefficient.

During the coding process, the validity of the coding was examined through the consistency of the two coders. The first coding consistency was 93.10%. For the documents with inconsistent coding, we finally reached an agreement after discussion. Accordingly, the basic information of the 28 target literature for meta-analysis were identified and coded as shown in Table 1.

**Table 1:** Basic information of the original study included in the analysis.

**References**	***N***	**Career stages**	**Teaching stages**	**Male%**	**Disciplines**	**Measurement tools**	**r**	**Dependent variable**
Mishne (2012)	32	In-service	Primary school	18%	General subject	TSE	0.32	TPCK
Wang (2014)	150	In-service	Middle school	50%	English	TISE	0.18	TPCK
Wang (2020)	226	Pre-service	University	19.03%	International Chinese	TISE	0.76	TPCK
Durak (2021)	401	In-service	Middle and primary school	41.90%	General subject	TISE	0.612	TPCK
Cai et al. (2019)	111	In-service	University	40%	General subject	CSE	0.55	TPCK
Yang (2018)	150	In-service	Middle school	50%	English	ICTSE	0.33	TPCK
Oskay (2017)	54	In-service	Middle and primary school	43%	General subject	CSE	0.486	TPCK
Tondeur et al. (2017b)	688	Pre-service	Middle and primary school	26.20%	General subject	ICTSE	0.65	TPCK
Abbitt (2011)	45	Pre-service	Preschool	4%	General subject	TISE	0.813	TPCK
Joo et al. (2018)	396	Pre-service	Middle school	35.50%	General subject	TSE	0.49	TPCK
Semiz and Ince (2012)	756	Pre-service	Middle and primary school	56.20%	Physical education	TISE	0.77	TPCK
López-Vargas et al. (2017)	208	In-service	Middle and primary school	22.10%	General subject	CSE	0.448	TPCK
Dong et al. (2020)	366	In-service	Middle and primary school	42.30%	General subject	CSE	0.62	TPCK
Song and Sun (2014)	340	In-service	Primary school	20.61%	English	TSE	0.56	TPCK
Wang and Zhao (2021)	298	Pre-service	Middle and primary school	36.58%	General subject	CSE	0.797	TPCK
Kaşci and Selçuk (2021)	1,127	In-service	Primary school	39.20%	General subject	TSE	0.76	TPCK
Zahwa et al. (2021)	27	Pre-service	Primary school	50%	Science	TISE	0.736	TPCK
Keser et al. (2015)	713	Pre-service	Middle and primary school	37%	General subject	TISE	0.779	TPCK
Sahin et al. (2013)	163	Pre-service	Middle and primary school	44%	General subject	CSE	0.58	TPCK
He (2013)	150	In-service	Middle school	27%	English	TISE	0.36	TPCK
Chen (2018)	110	In-service	Middle school	13.60%	English	TISE	0.786	TPCK
Cao (2016)	255	Pre-service	Middle and primary school	50%	Mathematics	TISE	0.728	TPCK
Chen (2021)	127	Pre-service	Middle school	18.10%	Chemistry	TISE	0.431	TPCK
Xu (2020)	281	Pre-service	Middle and primary school	11.40%	English	TISE	0.574	TPCK
Karalar and Altan (2016)	271	Pre-service	Primary school	28.80%	General subject	TSE	0.55	TPCK
Ariani (2015)	166	In-service	Primary school	50%	Mathematics	TISE	0.403	TPCK
Nathan (2009)	100	Pre-service	Middle and primary school	94%	General subject	TISE	0.42	TPCK
Bakar et al. (2020)	66	In-service	Middle school	15.20%	Mathematics	TISE	0.733	TPCK

(1) In order to save space, only the first author is listed in the table; (2) The proportion of men is 50%, indicating that there is no gender distinction in the literature; (3) General subject refers to the subjects that are not distinguished.

### Data analysis

In this study, CMA3.0 (Comprehensive Meta-analysis 3.0) was used for meta-analysis, and the correlation coefficient r was used as the effect size. In the coding process, some literature do not directly report the correlation coefficient between information technology integration self-efficacy and TPACK, but report the *F*-value, *t*-value, or *X*^2^-value. We adopted the formula of Wang and other scholars (Wang et al., 2013) to convert it into r value. The applicable condition of fixed effects model is that we assumed all test results have the same and true effect size, and the comprehensive effect is estimate of this common effect size. In contrast, we assumed the true effect size of each study is different in random effects model, and the comprehensive effect is estimate of the mean of the effect size. Specifically, if the total effect size of the meta-analysis is only for the study population and does not generalize to other populations, the fixed effects model should be used; on the contrary, if the subject groups, measurement tools, and experimental paradigms of the meta-analysis literature are different, these differences will affect the final results, and it is reasonable to use random effects model in this case (Wu and Liu, 2014). This study pressed for explore the moderating effect of each variable, therefore, using random effects model is in line with the actual situation.

## Results

### Homogeneity test

According to Higgins' classification standard of *I*^2^, 25, 50, and 75% represent low, medium, and high degree of heterogeneity, respectively (Higgins et al., 2003). The results of the heterogeneity test (Q = 429.03, *I*^2^ = 93.71, *p* < 0.001) proved that 93.71% of the observed variation is owing to real differences in this relationship between information technology integration self-efficacy and TPCK. The Tau-squared value was 0.056, indicating that 5.6% of the variation between studies could be used to calculate the weight. When the effect sizes are heterogeneous, random effects model is usually used for meta-analysis, which is consistent with the previous inferences.

### Assessment of publication bias

**Figure 2:**
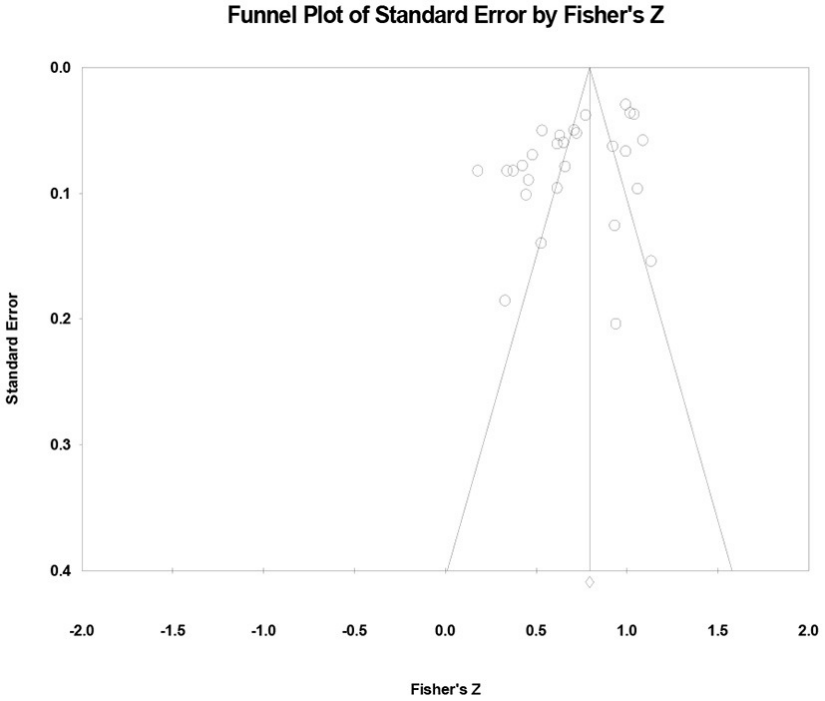
Funnel plot of effect sizes of the correlation between information technology integration self-efficacy and TPCK.

It can be seen from the funnel plot (Figure 2) that the distribution of literature selected by meta-analysis is basically symmetrical. And there are few points at the bottom right of the funnel plot, which represented that there are few studies with large effect size and poor accuracy; in addition, most of the points are concentrated at the top of the funnel plot, and the points are concentrated at the vertex, indicating that the error is small and the sample size is large. Consequently, the meta-analysis of this study is little affected by publication bias.

Because the funnel plot may be subjective, the Begg and Mazumdar's rank correlation test is further used to evaluate the publication bias. The test results demonstrated that Kendall's Tau value is −0.196 (*p* > 0.05), indicating that there is no publication bias in this meta-analysis, which is consistent with the funnel plot results.

### Main effect test

The random effect model was used to test the effect size of the literature that met the requirements. This meta-analysis includes 28 independent samples, with a total of 7,777 subjects. The results of random effect model manifested that the correlation coefficient between teachers' information technology integration self-efficacy and TPACK is 0.607 (CI = 0.545–0.663, Z = 14.776, *p* < 0.001), indicating that teachers' information technology integration self-efficacy and TPACK have a moderate positive correlation.

### Moderating effect test

This study examined the moderating effects of subjects' gender, career stages, teaching stages, disciplines, and measurement tools on the relationship between teachers' information technology integration self-efficacy and TPACK.

The results in Table 2 showed that the career stage of the subjects (Qb = 4.296, *P* < 0.05) affects the relationship between teachers' information technology integration self-efficacy and TPCK. Specifically, the correlation coefficient between pre-service teachers' information technology integration self-efficacy and TPACK is 0.666, which is significantly higher than that of in-service teachers. Gender (Qb = 2.448, *P* > 0.05), teaching stages (Qb = 7.118, *P* > 0.05), disciplines (Qb = 8.370, *P* > 0.05), and measurement tools (Qb = 1.140, *P* > 0.05) of the participants, all these above have no moderating role on the relationship between teachers' information technology integration self-efficacy and TPACK.

**Table 2:** The moderating effect of the relationship between teachers' information technology integration self-efficacy and TPACK.

**Moderator**	**Category**	**k**	**r**	**95% CI**	**Qb(df)**	***p***
Male proportion	0–20%	7	0.666	0.539, 0.763	2.448(3)	0.485
	21–40%	10	0.619	0.512, 0.706		
	41–60%	10	0.568	0.447, 0.668		
	81–100%	1	0.42	−0.074, 0.749		
Career stages	pre-service	14	0.666	0.594, 0.727	4.296(1)	0.038
	In-service	14	0.538	0.425, 0.635		
Teaching stages	Preschool	1	0.813	0.542, 0.931	7.118(4)	0.13
	Primary school	6	0.581	0.436, 0.696		
	Middle school	7	0.497	0.353, 0.618		
	University	2	0.673	0.453, 0.815		
	Middle and primary school	12	0.645	0.561, 0.715		
Disciplines	Mathematics	3	0.639	0.45, 0.773	8.37(6)	0.212
	English	6	0.493	0.338, 0.622		
	International Chinese	1	0.76	0.494, 0.896		
	Physical education	1	0.77	0.522, 0.898		
	Chemistry	1	0.431	−0.009, 0.731		
	Science	1	0.736	0.337, 0.911		
	General subject	15	0.619	0.54, 0.688		
Measuring tools	TISE	15	0.634	0.542, 0.711	1.140(3)	0.767
	ICTSE	2	0.513	0.190, 0.736		
	CSE	6	0.599	0.439, 0.722		
	TSE	5	0.568	0.382, 0.709		

## Discussion

### Relationship between teachers' information technology integration self-efficacy and TPACK

This study conducted a meta-analysis of the empirical research on the relationship between teachers' information technology integration self-efficacy and TPACK in recent 16 years, including 28 studies and 7,777 subjects. The results showed that teachers' information technology integration self-efficacy is significantly positively correlated with TPACK (r = 0.607, *p* < 0.001), indicating that teachers' information technology integration self-efficacy is closely related to TPACK. As one of the important contributing factors of teaching reform, technology has been confirmed by many studies. Teachers' belief in the function of information technology affects the teaching practice of integrating information technology (Abbitt, 2011; Anderson et al., 2011); teachers' belief in information technology ability also affects teachers' attitude toward using information technology in teaching (Papastergiou, 2010; Rohaan et al., 2012). At the same time, teachers who believe in the usefulness of technology are more willing to integrate technology into the classroom, which will have a positive impact on learning results (Nathan, 2009; Karataş, 2014). For instance, the positive impact of TPACK on student academic achievement has been verified by multiple meta-analysis studies (Young, 2016). Bandura held the opinion that the conjunction of interactions between people and context is a key idea in social cognitive theory (Bandura, 2001). In view of the individual factors of teachers and the complex education system, we believe that the improvement of teachers' information technology integration self-efficacy has higher requirements for the information technology environment at the school level, and in turn, it also affects the information technology environment at school. So, the positive relationship between teachers' information technology integration self-efficacy and TPACK should be highly valued by educational practitioners. We can attempt to improve teachers' TPACK level through the improvement of teachers' information technology integration self-efficacy.

Besides, this study further verified many previous research conclusions, that is, teachers' information technology integration self-efficacy is closely related to teachers' technology use behavior. Teachers' technology self-efficacy affects their technology integration behavior in the classroom (Compeau and Higgins, 1995; Albion, 1999). The experience of using technology inside and outside the classroom promotes teachers to establish their faith in the function and value of technology (Lumpe and Chambers, 2001). The higher the ICT self-efficacy level pre-service teachers have, the more frequent information technology integration behaviors they generate (Kavanoz et al., 2015). In classroom teaching, teachers with higher ICT self-efficacy tend to show more positive emotions in the process of information technology integration (Moreira-Fontán et al., 2019). TPACK, as the representation of teachers' information technology integration in teaching practice behaviors, its importance should also arouse the attention of educational practitioners and managers. Although TPACK is a compound concept, after the continuous in-depth theoretical and practical research, it has reached a consensus in academia: The professional knowledge related to a successful subject specific integration of technology is commonly subsumed under the concept of technological pedagogical content knowledge. Accordingly, the further clarification of the relationship between information technology integration self-efficacy and TPACK in this study provided a clear operational path for teachers' professional development.

### Moderating effect of the relationship between teachers' information technology integration self-efficacy and TPACK

#### The moderating role of gender

The results of inter group difference analysis showed that gender of the subjects did not affect the relationship between teachers' information technology integration self-efficacy and TPACK, and there was no significant moderating effect (Qb = 2.448, *P* > 0.05). This study is divided into four groups according to the male proportion. There is a significant positive correlation between teachers' information technology integration self-efficacy and TPACK in the group. This correlation gradually decreases with the increase of the male proportion between groups, and the difference is not statistically significant, but this result should be paid attention to. The conclusion of this meta-analysis is consistent with that of some previous studies. There is no gender difference in teachers' information technology integration self-efficacy (Keser et al., 2015), at the same time, there is no gender difference in TPACK (Redmond and Peled, 2019). Therefore, the test of the moderating effect of gender on the relationship between teachers' information technology integration self-efficacy and TPACK is worth further discussion in the follow-up research. We can try to divide male and female into groups to verify the moderating effect of gender more directly, so as to come to scientific research conclusions.

#### The moderating role of career stages

The results of inter group difference analysis showed that the career stages affected the link between teachers' information technology integration self-efficacy and TPACK, and there was a significant moderating effect (Qb = 4.296, *P* < 0.05). Although the relationship between teachers' information technology integration self-efficacy and TPACK in pre-service and in-service stages reached a significant level, the pre-service teachers group was significantly higher than in-service teachers group. The reason for this moderating effect may lie in the differences in their cognition and action of information technology integration. Under the digital background, the integration of technology and teaching is an important measure for teachers to cultivate students' ability to cope with the digital future. Thus, it is generally argued that pre-service teachers should acquire subject-specific professional knowledge regarding technology integration to support their future students' learning. In this regard, some scholars have found that pre-service teachers should develop adequate motivational orientations (e.g., self-efficacy) (Backfisch et al., 2020). Despite the potential of integrating technology for teaching, however, research has demonstrated that in many educational systems teachers rarely adopt technology into teaching (Fraillon et al., 2020). From this point of view, it may be explained that the correlation coefficient in the pre-service teacher group is higher than that of in-service teacher group. The confirmation of this result also enlightens us to focus on the information technology integration self-efficacy and TPACK level of in-service teachers.

#### The moderating role of measurement tools

The results of inter group difference analysis revealed that the measurement tools had no effect on the relationship between teachers' information technology integration self-efficacy and TPACK, and had no significant moderating effect (Qb = 1.140, *P* > 0.05). It is inconsistent with our hypothesis. This result further illustrated the stability of the relationship between teachers' information technology integration self-efficacy and TPACK, which is not affected by objective research conditions and other factors. As the previous literature review showed, the measurement tools of teachers' information technology integration self-efficacy in this study are divided into three types, including four scales. The TISE measurement tool developed by Wang has specific measurement content, examples are as follows, “I feel confident that I have the skills necessary to use the computer for instruction” “I feel confident I can mentor students in appropriate uses of technology.” Computer self-efficacy (CSE) reflects teachers' information technology integration self-efficacy through the measurement of teachers' belief in the ability to use computers in the teaching process, which is also reasonable. In addition, the classic teacher self-efficacy scale is used to measure, and the meta-analysis literature included all emphasizes the self-efficacy in the context of information technology integration. Although it is not a direct measurement tools, it still has strong pertinence. In a word, the measurement tools of teachers' information technology integration self-efficacy have no moderating effect, which proves that the measurement tools and methods in this study are scientific, and provides an important reference for subsequent research.

### The moderating role of teaching stages and disciplines

The results of inter group difference analysis illustrated that the participants' teaching stages (Qb = 7.118, *P* > 0.05) and teaching subjects (Qb = 8.370, *P* > 0.05) had no moderating effect on the link between teachers' information technology integration self-efficacy and TPACK. This result is inconsistent with our hypothesis. However, we should be cautious about this conclusion. In terms of the literature included in this meta-analysis, there are only 1 and 2 papers in preschool and university, respectively; at the same time, teaching subjects are also relatively scattered, among which chemistry, physical education, science and international Chinese are all included in only one paper, and there are 15 articles that do not distinguish disciplines. Nevertheless, this meta-analysis also gave an empirical research conclusion. However, it is worth noting that teaching is a complex practical activity. In specific and special teaching situations, the effectiveness of TPACK mostly depends on the compatibility between teachers and Context in teaching (Herring et al., 2008). The value of TPACK lies in instructing teachers how to promote teaching. TPACK has different representations in different subjects and backgrounds, only by conducting refined research on it from the perspective of various subjects can teachers improve their TPACK level in a targeted manner.

## Limitations

First, although this study tries to broaden the ways of collecting literature and increase the types of publications to avoid publication bias, as a result of language constraints, only Chinese and English literature are searched, and there are still documents that meet the selection criteria that are not included in the meta-analysis. Second, in the analysis of moderating effect, the sample distribution of some moderating variables is not balanced, and some moderating variable subgroups only include several independent studies, which may affect the analysis of moderating effect. For example, there are few studies on the stage of preschool and university. Third, the moderating effect of subjects' age was not tested. At present, most studies on the age effect of TPACK have confirmed that there is a negative correlation. Song showed that young teachers under the age of 25 have advantages in TK and TPK compared with teachers of other age groups; López-Vargas found that teachers' TK, TCK, PK were also negatively correlated with age; Li and Cai found that TPACK of Taiwan in-service teachers based on online teaching was negatively correlated with age and teaching age (Lee and Tsai, 2010). Another study showed that age was only negatively correlated with technology-related TPACK dimensions, such as TK, TCK, TPK and TPCK (Liang et al., 2013; Koh et al., 2014). Chen found that teachers' TK was negatively correlated with age, while TCK, TPK, TPCK were not correlated with age. There are also individual studies that show that all dimensions of teachers' TPACK have nothing to do with teachers' age (He, 2013; Wang, 2014). Then, it remains to be studied whether age has a moderating effect on the relationship between teachers' information technology integration self-efficacy and TPACK.

## Conclusions

Using the method of meta-analysis, this study found that there was a moderate positive correlation between teacher' information technology integration self-efficacy and TPACK. The relationship between them was moderated by the subjects' career stages, but not by gender, disciplines, teaching stages and measurement tools.

In terms of theoretical significance, based on previous research conclusions, this paper systematically confirmed the relationship between teachers' information technology integration self-efficacy and TPACK through meta-analysis. It is an expansion of theoretical research in both the field of teacher education research and educational information technology research, which helps theoretical researchers gain a more general and scientific understanding of the relationship between them. To be specific, the contribution of theoretical research is from the perspective of examining the moderating effect of related factors, to explore the influence of gender, measurement tools, career stages, disciplines and teaching stages. At present, such research literature is still rare, which lays a certain foundation for future related research.

As for practical significance, in the digital era of information technology involvement in education and teaching reform, teachers' information technology knowledge and competency is related to teachers' professional development practice, and it also determines the quality of education and teaching. TPACK is increasingly valued by education practitioners. The conclusion drawn in this study provides an empirical reference for the decision-making of teacher education practitioners, and also offers a specific operational direction for the professional development of pre-service teachers and in-service teachers. For example, in order to enhance self-efficacy of teacher information technology integration, education administrators can organize teacher training and formulate daily management systems. At the same time, teachers should also strengthen their own self-efficacy of information technology integration and improve their own TPACK literacy level based on their own actual professional development.

## Data availability statement

The original contributions presented in the study are included in the article/supplementary material, further inquiries can be directed to the corresponding author.

## Author contributions

YZ and SL: conceptualization and validation. YZ and YW: methodology. YZ: formal analysis, writing—original draft preparation, visualization, supervision, and funding acquisition. YW and SL: writing—review and editing. All authors have read and agreed to the published version of the manuscript.

## Funding

This research was funded by Social Science Project of Education Department of Liaoning Province (LJKQR20222507), Social Science Planning Fund Education Project of Liaoning Province (Grant No. L20CED006), and Project of Social Science Foundation of Xinjiang Uygur Autonomous Region (Grant No. 22CMZ018).

## Conflict of interest

The authors declare that the research was conducted in the absence of any commercial or financial relationships that could be construed as a potential conflict of interest.

## Publisher's note

All claims expressed in this article are solely those of the authors and do not necessarily represent those of their affiliated organizations, or those of the publisher, the editors and the reviewers. Any product that may be evaluated in this article, or claim that may be made by its manufacturer, is not guaranteed or endorsed by the publisher.
